# Supplementary material for: Deletion of wfs1 Impairs Oligodendrocyte Precursor Cells Dorsal Distribution and Myelination Through the wfs1-hmgcs1 Axis in Zebrafish
Source: Biology (Basel). 2026 Aug 16;15(16):1402. doi: 10.3390/biology15161402 (PMC13510151; doi:10.3390/biology15161402)
Supplement: Supplementary file 1 [file biology-15-01402-s001.zip › File S1-Transcriptome sequencing data and differentially expressed genes (DEGs) in wfs1-- zebrafish at 5 dpf/2.KEGG/wfs1KOvsWT/GSEA/WT/AMINOACYL_TRNA_BIOSYNTHESIS/AMINOACYL_TRNA_BIOSYNTHESIS(DRE00970).html]

Details for gene set AMINOACYL\_TRNA\_BIOSYNTHESIS(DRE00970)[GSEA]

|  || Dataset | deg.sample |
| Phenotype | sample.cls |
| Upregulated in class | WT |
| GeneSet | AMINOACYL\_TRNA\_BIOSYNTHESIS(DRE00970) |
| Enrichment Score (ES) | -0.2751026 |
| Normalized Enrichment Score (NES) | -1.1068232 |
| Nominal p-value | 0.35756385 |
| FDR q-value | 0.5198102 |
| FWER p-Value | 1.0 |
Table: GSEA Results Summary

  

Fig 1: Enrichment plot: AMINOACYL\_TRNA\_BIOSYNTHESIS(DRE00970)      
 Profile of the Running ES Score & Positions of GeneSet Members on the Rank Ordered List

  

| PROBE | DESCRIPTION (from dataset) | GENE SYMBOL | GENE\_TITLE | RANK IN GENE LIST | RANK METRIC SCORE | RUNNING ES | CORE ENRICHMENT || 1 | 101884223 | LOC101884223 |  |  | 682 | 2.621 | 0.0594 | No |
| 2 | 792787 | aars2 |  |  | 1290 | 1.819 | 0.0965 | No |
| 3 | 110439041 | LOC110439041 |  |  | 1389 | 1.733 | 0.1469 | No |
| 4 | 324940 | aars |  |  | 3179 | 0.997 | 0.1214 | No |
| 5 | 368235\_1 | yars |  |  | 3337 | 0.966 | 0.1463 | No |
| 6 | 394154 | sars2 |  |  | 3743 | 0.880 | 0.1607 | No |
| 7 | 503745 | wars2 |  |  | 5791 | 0.608 | 0.1152 | No |
| 8 | 571144 | dars2 |  |  | 6175 | 0.570 | 0.1207 | No |
| 9 | 445405 | sars |  |  | 9661 | 0.323 | 0.0212 | No |
| 10 | 493608 | farsb |  |  | 9916 | 0.309 | 0.0228 | No |
| 11 | 569778 | larsb |  |  | 10072 | 0.299 | 0.0272 | No |
| 12 | 100001219 | iars2 |  |  | 10408 | 0.278 | 0.0252 | No |
| 13 | 100330276 | tarsl2 |  |  | 11191 | 0.233 | 0.0078 | No |
| 14 | 561673 | nars2 |  |  | 11243 | 0.231 | 0.0134 | No |
| 15 | 558898 | si:rp71-1c23.3 |  |  | 12189 | 0.180 | -0.0108 | No |
| 16 | 556702 | yars2 |  |  | 12956 | 0.143 | -0.0304 | No |
| 17 | 562037 | eprs |  |  | 13491 | 0.116 | -0.0436 | No |
| 18 | 449661\_1 | tars |  |  | 14999 | 0.046 | -0.0895 | No |
| 19 | 541317\_2 | gatb |  |  | 15725 | 0.010 | -0.1120 | No |
| 20 | 368284 | cars |  |  | 16791 | -0.026 | -0.1446 | No |
| 21 | 393123 | sepsecs |  |  | 17049 | -0.034 | -0.1516 | No |
| 22 | 793097 | fars2 |  |  | 19976 | -0.165 | -0.2384 | No |
| 23 | 114427 | vars |  |  | 20251 | -0.177 | -0.2416 | No |
| 24 | 108179007 | wars |  |  | 21182 | -0.225 | -0.2639 | No |
| 25 | 751732 | zgc:153247 |  |  | 21541 | -0.243 | -0.2676 | Yes |
| 26 | 100170827 | vars2 |  |  | 21776 | -0.256 | -0.2671 | Yes |
| 27 | 100333765 | tars2 |  |  | 21843 | -0.259 | -0.2611 | Yes |
| 28 | 334393 | iars |  |  | 21866 | -0.260 | -0.2538 | Yes |
| 29 | 393589 | rars2 |  |  | 22438 | -0.292 | -0.2627 | Yes |
| 30 | 325494 | pars2 |  |  | 22715 | -0.308 | -0.2619 | Yes |
| 31 | 556680 | mtfmt |  |  | 22853 | -0.316 | -0.2564 | Yes |
| 32 | 566565\_2 | mars2 |  |  | 22951 | -0.324 | -0.2495 | Yes |
| 33 | 338183 | mars |  |  | 22995 | -0.327 | -0.2408 | Yes |
| 34 | 337230 | gars |  |  | 23065 | -0.332 | -0.2327 | Yes |
| 35 | 449661\_2 | tars |  |  | 23227 | -0.343 | -0.2272 | Yes |
| 36 | 280647 | kars |  |  | 23666 | -0.371 | -0.2295 | Yes |
| 37 | 337070 | rars |  |  | 23766 | -0.378 | -0.2209 | Yes |
| 38 | 393745 | wars |  |  | 23955 | -0.389 | -0.2148 | Yes |
| 39 | 568332 | nars |  |  | 25266 | -0.464 | -0.2416 | Yes |
| 40 | 100126021 | lars2 |  |  | 25322 | -0.468 | -0.2289 | Yes |
| 41 | 555187 | cars2 |  |  | 25403 | -0.475 | -0.2168 | Yes |
| 42 | 447847 | hars |  |  | 26438 | -0.571 | -0.2317 | Yes |
| 43 | 541317 | gatb |  |  | 27561 | -0.680 | -0.2459 | Yes |
| 44 | 327329 | qrsl1 |  |  | 27700 | -0.701 | -0.2286 | Yes |
| 45 | 324025 | dars |  |  | 28061 | -0.751 | -0.2168 | Yes |
| 46 | 541317\_1 | gatb |  |  | 28709 | -0.860 | -0.2106 | Yes |
| 47 | 792787\_1 | aars2 |  |  | 28790 | -0.876 | -0.1861 | Yes |
| 48 | 692329 | farsa |  |  | 29306 | -1.002 | -0.1713 | Yes |
| 49 | 103911967 | LOC103911967 |  |  | 30478 | -1.493 | -0.1620 | Yes |
| 50 | 394188 | qars |  |  | 30725 | -1.703 | -0.1173 | Yes |
| 51 | 324940\_1 | aars |  |  | 31889 | -4.987 | 0.0000 | Yes |
Table: GSEA details [plain text format]

  

Fig 2: AMINOACYL\_TRNA\_BIOSYNTHESIS(DRE00970)      
 Blue-Pink O' Gram in the Space of the Analyzed GeneSet

  

Fig 3: AMINOACYL\_TRNA\_BIOSYNTHESIS(DRE00970): Random ES distribution      
 Gene set null distribution of ES for **AMINOACYL\_TRNA\_BIOSYNTHESIS(DRE00970)**
